# Weighted gene co-expression network analysis identifies genes related to HG Type 0 resistance and verification of hub gene *GmHg1*

**DOI:** 10.3389/fpls.2022.1118503

**Published:** 2023-01-27

**Authors:** Haipeng Jiang, Changjun Zhou, Jinglin Ma, Shuo Qu, Fang Liu, Haowen Sun, Xue Zhao, Yingpeng Han

**Affiliations:** ^1^ Key Laboratory of Soybean Biology in Chinese Ministry of Education (Key Laboratory of Soybean Biology and Breeding/Genetics of Chinese Agriculture Ministry), Northeast Agricultural University, Harbin, China; ^2^ Soybean Molecular Breeding Faculty Daqing Branch, Heilongjiang Academy of Agricultrual Science, Daqing, China

**Keywords:** soybean cyst nematode, WGCNA, hub gene, protein kinase, *GmHg1*

## Abstract

**Introduction:**

The soybean cyst nematode (SCN) is a major disease in soybean production thatseriously affects soybean yield. At present, there are no studies on weighted geneco-expression network analysis (WGCNA) related to SCN resistance.

**Methods:**

Here, transcriptome data from 36 soybean roots under SCN HG Type 0 (race 3) stresswere used in WGCNA to identify significant modules.

**Results and Discussion:**

A total of 10,000 differentially expressed genes and 21 modules were identified, of which the module most related to SCN was turquoise. In addition, the hub gene GmHg1 with high connectivity was selected, and its function was verified. GmHg1 encodes serine/threonine protein kinase (PK), and the expression of GmHg1 in SCN-resistant cultivars (‘Dongnong L-204’) and SCN-susceptible cultivars (‘Heinong 37’) increased significantly after HG Type 0 stress. Soybean plants transformed with GmHg1-OX had significantly increased SCN resistance. In contrast, the GmHg1-RNAi transgenic soybean plants significantly reduced SCN resistance. In transgenic materials, the expression patterns of 11 genes with the same expression trend as the GmHg1 gene in the ‘turquoise module’ were analyzed. Analysis showed that 11genes were co-expressed with GmHg1, which may be involved in the process of soybean resistance to SCN. Our work provides a new direction for studying the Molecular mechanism of soybean resistance to SCN.

## 1 Introduction

The soybean cyst nematode (SCN), a worldwide soybean disease caused by soil-borne plant parasitic nematodes, can generally reduce soybean yields by 30–50%; however, in severe cases, they can cause complete loss (Cook et al., 2012). At present, SCN directly damages more than one million hectares of land in China’s soybean-producing areas, causing direct economic losses of up to 20 billion yuan per year (Cook et al., 2012). These losses have driven the development of several technologies aimed at combating SCN to reduce associated yield losses.

Many years of research and practice have shown that reasonable crop rotation, biological control, and chemical use can control SCN to some extent, but breeding soybean varieties resistant to SCN is the most economical, safe, and effective way to control this disease (Han et al., 2017). The *rhg1* locus from SCN-resistant soybean plant introgression ‘PI 88788’ and the *Rhg4* locus from ‘Peking (PI 548402)’ are the main sources of resistance used in commercial varieties to reduce yield losses in fields infested with SCN (Mitchum, 2016). However, over-reliance on a small number of resistance sources (particularly PI 88788 and Peking) has resulted in SCN populations gradually overcoming this resistance in the field (Colgrove and Niblack, 2008; Wu et al., 2009; Jiao et al., 2015). Therefore, it is necessary to continuously identify new resistance sources and genes to control SCN more effectively.

When plants are disturbed by external pests and diseases, gene expression is re-coded at the transcriptional, post-transcriptional, and post-translational levels in response to external interference to produce defense mechanisms (Alkharouf et al., 2006; Ithal et al., 2007a; Ithal et al., 2007b). With the rapid development of second-generation sequencing in recent years, the application of second-generation sequencing technologies in transcriptome sequencing has become increasingly common (Bhattacharyya et al., 2013). Because of the advantage of sequencing depth, RNA sequencing (RNA-Seq) is a better method for revealing individual gene expression in a specific time and tissue and has been widely used for SCN (Barakat et al., 2009; Dassanayake et al., 2009; Bhattacharyya et al., 2013; Jain et al., 2016). A range of cell wall repair genes, defense genes (PPR and NLRs), MAPK (mitogen-activated protein kinase), WRKY and MYB transcription factors (TF), heat shock protein (HSP) genes, PR genes, and phenanthrene metabolism genes have been identified based on microarray and RNA-Seq transcriptome analysis (Klink et al., 2007; Puthoff et al., 2007; Kandoth et al., 2011; Mazarei et al., 2011; Wan et al., 2015; Kang et al., 2018; Neupane et al., 2019; Song et al., 2019; Jiang et al., 2020; Miraeiz et al., 2020). Some of these genes are differentially expressed genes (DEGs) among resistant and susceptible varieties, and some are SCN-stress responsive genes. However, there are many screened differential genes associated with SCN, and it is time-consuming and laborious to validate them all; therefore, further screening of the differential genes screened by transcriptome sequencing is particularly important.

Weighted gene correlation network analysis (WGCNA) is a bioinformatics algorithm used to describe the correlation patterns of gene expression. It relies on easy-to-understand statistical methods and improvements to simple correlation networks (Zhang and Horvath, 2005). WGCNA was developed to more efficiently analyze microarray datasets by quantifying not only the correlation between individual gene pairs but also the extent to which these genes share the same neighbors (Langfelder and Horvath, 2008). WGCNA uses systems biology to find similarities in gene expression, cluster genes with highly related expression into a module, obtain biologically significant co-expression modules, and screen the core gene (hub genes) (Langfelder and Horvath, 2008). Compared with other co-expression analysis methods, it uses the soft threshold approach to improving the sensitivity of module recognition (Wang et al., 2014; Giulietti et al., 2016). WGCNA has been applied to many crops. Tai et al. (2018) identified 35 co-expression modules, 20 of which were related to the synthesis of catechin, theanine, and caffeine, from which core genes regulating the metabolism of these three substances were obtained by analyzing the transcriptome data of different tissues of tea (*Camellia sinensis*) by WGCNA. Qi et al. (2018) identified 7,482 DEGs and 45 expression pattern clusters using paired comparison and K-means cluster analysis; 46 DEGs pattern modules were revealed by WGCNA analysis, and 7 hub genes involved in soybean oil and seed storage protein accumulation were identified. Therefore, the construction of high-throughput sequencing and gene co-expression networks are effective ways to rapidly identify the key regulatory factors in pathways associated with target traits. However, this has not been reported in the transcriptome sequencing of SCN-infected plants.

In China, the main physiological races of SCN causing serious economic loss of soybean are HG Type 2.5.7, HG Type 0 and HG Type 1.2.3.5.7, among which HG Type 0 is the most widely distributed (Han et al., 2017). We previously analyzed the transcriptome sequencing results of the SCN-resistant cultivars ‘Dongnong L-10’ and ‘Dongnong L-204’ and the SCN-susceptible cultivars ‘HN37’ after HG Type 0 stress and predicted that MAPK signaling cascades, transcription factors (AP2/EREBP, WRKY, MYB, NAC, bHLH, and C2H2), and plant hormone signal transduction pathways (jasmonic acid and salicylic acid pathway) may be involved in the response of soybean to HG Type 0 (Jiang et al., 2020; Jiang et al., 2021). To further screen for HG Type 0-related hub genes, we used transcriptome sequencing data obtained in the previous stage to mine the core genes associated with SCN resistance using WGCNA. We performed bioinformatic analysis and expression pattern analysis of core genes and constructed overexpression and RNAi interference materials for gene SCN-resistance identification, which helped to clarify gene function in depth and provided a good basis for further research to identify SCN-resistance mechanisms in this study.

## 2 Materials and methods

### 2.1 Transcriptome data

The RNA-Seq data of 36 soybean root samples treated with HG Type 0 stress were obtained from our previous study (Jiang et al., 2020; Jiang et al., 2021). ‘Heinong 37’ (developed by Heilongjiang Agricultural Academy, susceptible to SCN HG type 0), ‘Dongnong L-10’, and ‘Dongnong L-204’ (provided by Northeast Agricultural University, resistant to SCN HG type 0) were planted in pots with a diameter of 13 cm in a greenhouse (day/night temperature: 27–28°C; relative humidity: 60–70%; day/night illumination time 16h/8h), collected and inoculated with SCN using the method described by Wan et al. (2015). A random complete block design (RCBD) was used with 3 replicates of 10 seedlings each. Root samples inoculated with 2000 SCN (J2) were collected and sequenced at 0, 3, 7, and 10 days after inoculation. The RNA-Seq data of these 36 samples included transcriptome data of 28,656 genes, from which 10,000 genes with an average transcriptome FPKM value greater than 2.5 were selected for co-expression network analysis.

### 2.2 Building a sample clustering tree

The good “Genes Samples” function in the R packet of WGCNA was used to eliminate the genes and samples with too many missing values, and the sample-level clustering-pruning graph method was used to eliminate outlier samples—that is, to remove outlier samples that were significantly higher than other samples (Giulietti et al., 2016). The hclust function in R was used to construct the clustering tree. The Pick-Soft threshold function was used to analyze the network topology information, and the appropriate β value was selected as the soft threshold to construct the network to make the network meet the scale-free topology characteristics. The topological difference value of the network was calculated, and the genes were divided using a hierarchical clustering method to generate the gene clustering tree. The gene module with a high degree of correlation was identified by the one-step construction method; 50 was set as the minimum number of genes in the module, and the threshold of cutting height was 0.25 ( (Giulietti et al., 2016)). The gene module was identified by the cutting tree method, and modules with high similarity were combined. The gene groups were named with different colors, which is convenient for distinguishing different gene modules in subsequent gene functional recognition and visual analysis. The WGCNA package (https://horvath.genetics.ucla.edu/html/CoexpressionNetwork/Rpackages/WGCNA/Tutorials/) in R was used to perform the analysis, as described previously (Mason et al., 2009). The genes of MM > 0.85 and GS > 0.6 in the key modules were introduced into Cytoscape using the MMC (Maximum Margin Criterion) algorithm, and the core genes were selected according to their weight and gene network regulation position.

### 2.3 Plant materials, growth conditions, and inoculation

The soybean variety ‘Dongnong 50’ (DN50, SCN-susceptible cultivars) was used as the WT and the background plant for genetic transformation. ‘Dongnong L-204’ and ‘Heinong 37’ were used as resistant and susceptible cultivars, respectively, for gene expression pattern analysis. The seeds of soybean were germinated in vermiculite and peat soil (1:1). Plants were grown under long-day conditions (16 h light/8 h dark) at 25 ± 1°C for routine maintenance. HG Type 0 was obtained from the Soybean Research Institute of Northeast Agricultural University and was isolated and purified for many generations. A randomized complete block design (RCBD) was utilized, with 3 replicates and 10 seedlings per replicate. Each seedling was inoculated with approximately 2000 second-stage juvenile nematodes (J2s). Accordingly, mock-inoculation with distilled water was also conducted for each line as a control. All treatments and controls were watered daily to maintain soil moisture and promote uniform infection throughout the root system. Both SCN-inoculated and mock-inoculated root samples were harvested at 0, 5, 10, and 15 day post inoculation (dpi). A randomized complete block design (RCBD) was utilized with three replicates and ten seedlings per replicate. The preparation of the SCN egg suspension and its identification by acid fuchsin staining were performed following Jiang et al. (2021).

### 2.4 RNA extraction, gene expression analysis, and bioinformatics analysis

The RNA extraction methods were described previously (Jiang et al., 2021). The extracted complete RNA was reverse transcribed into cDNA. DEGs were selected and verified by qRT-PCR based on SYBR Green PCR Master Mix (TIANGEN BIOTECH, BeiJing, China) and the 7500 Fast PCR detection system. The relative mRNA level of each candidate gene was evaluated against soybean *GmACTIN* (GenBank Accession Number AF049106) as a reference gene. Three technical replicates were performed per gene, and the relative levels of transcript abundance were calculated using the 2^-ΔΔCT^ method (Jain et al., 2016). The sequences of the primer pairs were used to amplify the candidate genes (**Table S4**). The promoter elements of the 1000 bp pro-GmHg1 sequence were analyzed using PlantCARE software (http://bioinformatics.psb.ugent.be/webtools/plantcare/html/).

### 2.5 Plasmid construction and transformation of soybean

We identified the Coding sequence (CDS) of the soybean *GmHg1* gene in the plant database (https://phytozome.jgi.doe.gov/pz/portal.html) and amplified the full-length CDS sequence of *GmHg1* from the developing seeds of ‘Dongnong L-204’ by RT-PCR. For construction of the recombinant vectors GmHg1-OX and GmHg1-GFP, 2130-bp CDS of *GmHg1* were amplified from the cDNA of ‘DN50’ by overlapping PCR. Then, the PCR products were ligated to the linear vectors pCAMBIA3300 and pCAMBIA1302 using a homologous recombination system (ClonExpress II One Step Cloning Kit, Vazyme, China). For the construction of GmHg1-RNAi, the specific sequences of the 498-bp sense and antisense fragments of the *GmHg1* gene were cloned into the RNA interference plant expression vector pFGC5941 through homologous recombination. According to this method (Zhao et al., 2018), the recombinant plasmid GmHg1-GFP was transformed into *Arabidopsis* protoplasts and analyzed under a fluorescence microscope (Leica, Germany). The recombinant plasmids GmHg1-OX and GmHg1-RNAi were transformed into *Agrobacterium rhizogenes* EHA105 according to a previously described method (Zhao et al., 2018), and transgenic soybean was obtained.

## 3 Results

### 3.1 Constructing the clustering tree of all samples

**Figure 1:**
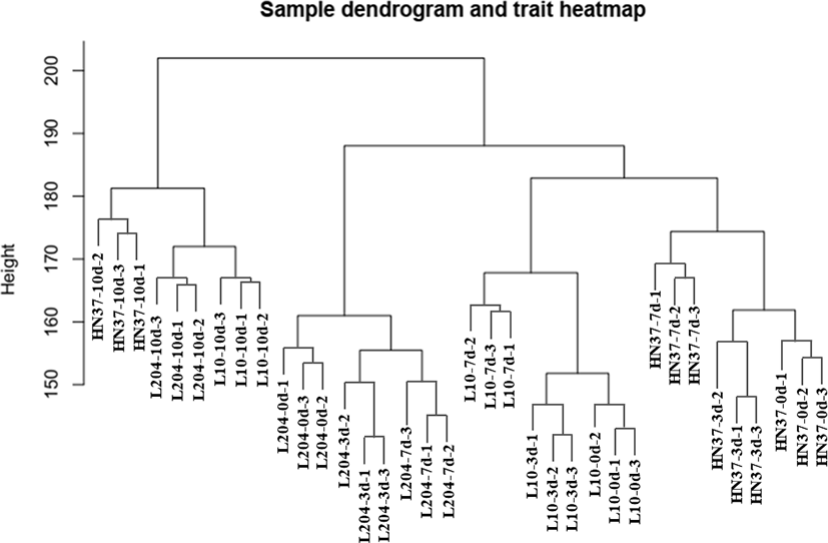
Cluster analysis diagram.

We used the roots of SCN-resistant cultivars (‘Dongnong L-10’, ‘Dongnong L-204’) and SCN-susceptible cultivars (‘Heinong 37’) under SCN HG type 0 stress for transcriptome sequencing, and 36 transcriptome samples were obtained. The transcriptome data of 36 samples were analyzed using WGCNA, and 10,000 genes with FPKM values greater than 2.5 were selected for soft threshold clustering. According to the expression trend, 10,000 genes were divided into 12 modules. Cluster analysis showed that the three biological repeat sequences of the samples were close to each other, and the overall effect was good (**Figure 1**).

### 3.2 Construction of a weighted gene co-expression network

**Figure 2:**
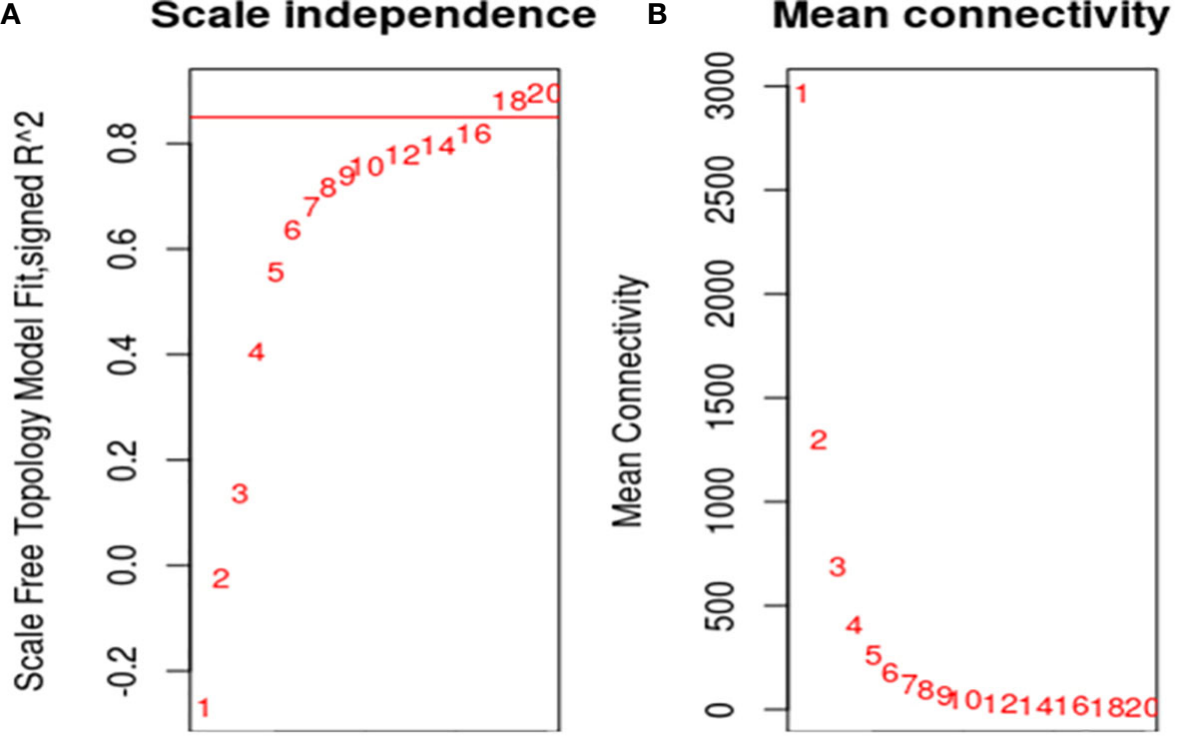
Screening of optimal soft threshold. **(A)**: The soft threshold was determined by the scale-free network index. R2 was set to 0.85, and the best soft threshold was 18; **(B)**: The soft threshold was determined by network connectivity. The average value of the gene adjacency coefficient in the gene network corresponding to different soft thresholds reflects the average network connection level. The network connectivity was better when the soft threshold was 18.

**Figure 3:**
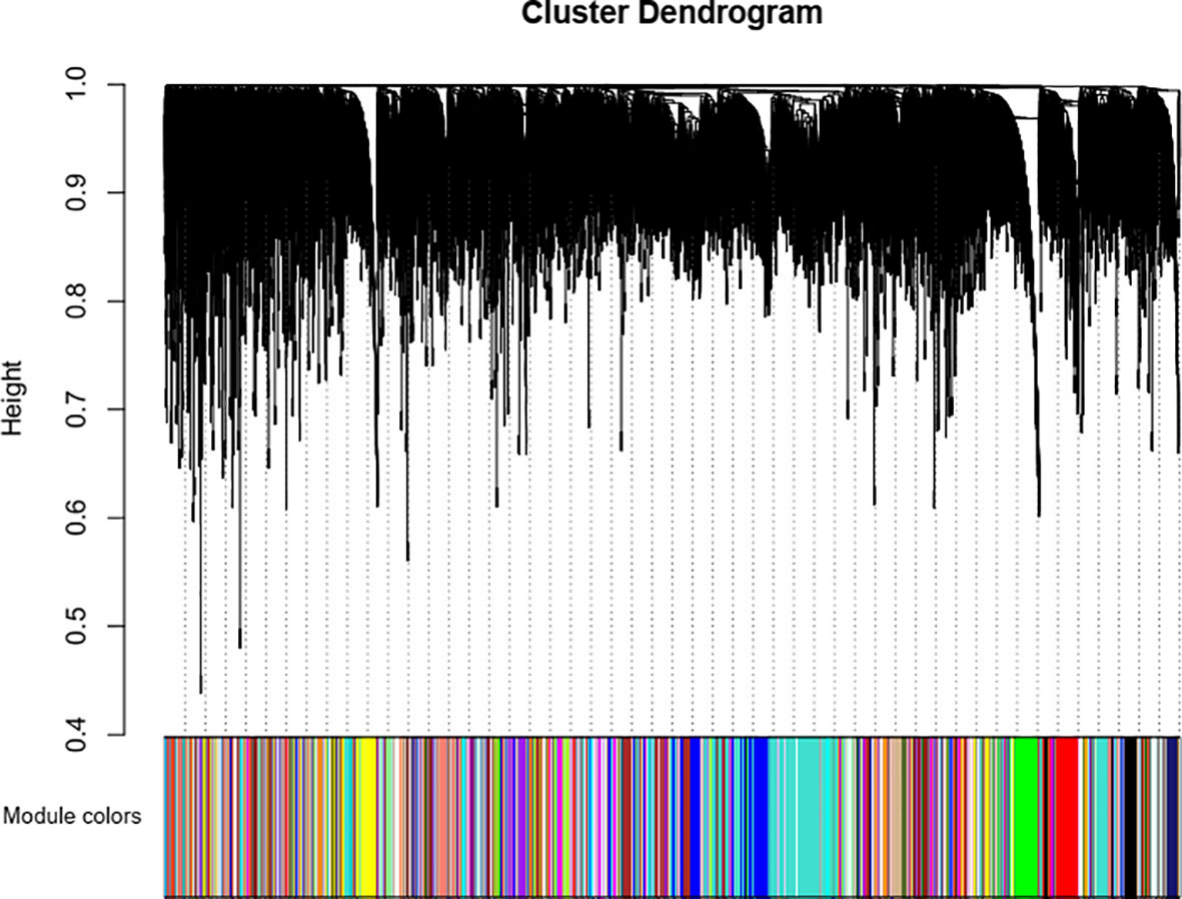
Identification of gene co-expression modules *via* hierarchical average linkage clustering. The color row underneath the dendrogram shows the module assignment determined by the dynamic tree cut.

**Figure 4:**
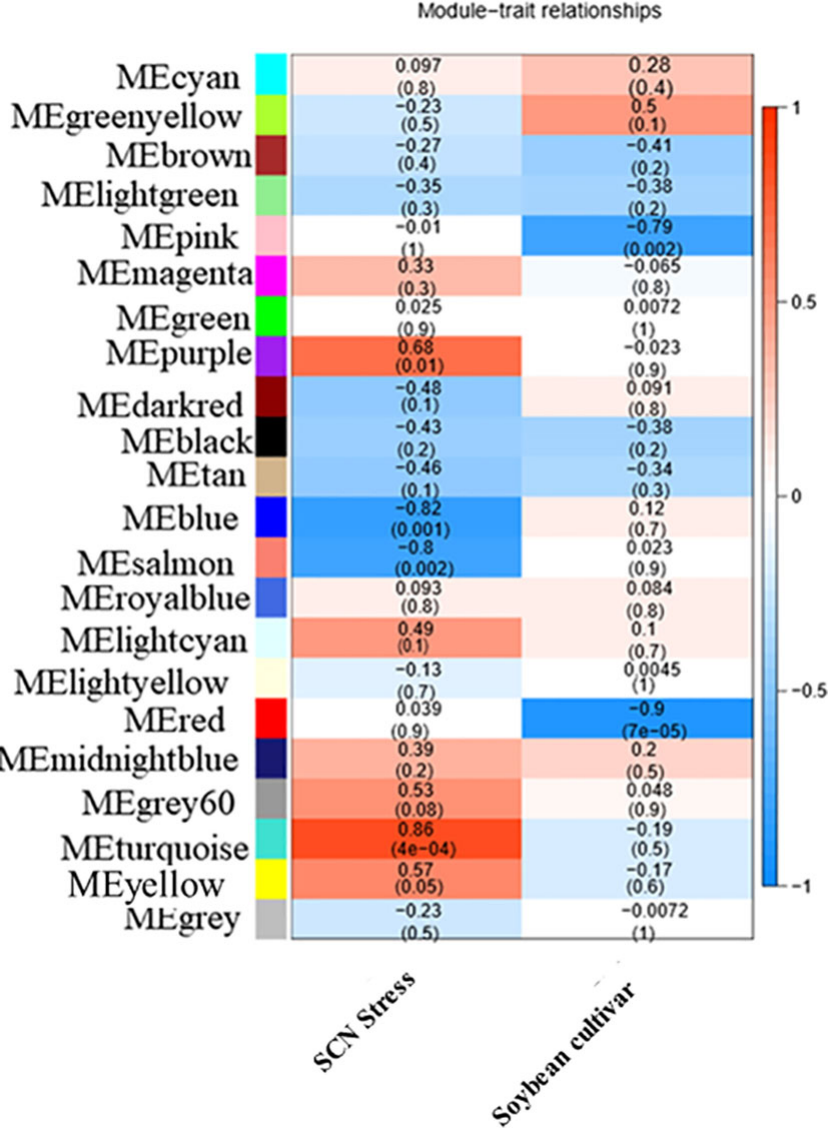
Heat map of the correlation between templates and traits. Each row represents a module eigengene, and each column represents a different characteristic. Each cell contained the corresponding correlation and p-value. The table is color-coded by correlation according to the color legend.

To ensure that it conforms to the scale-free network distribution, the WGCNA needs to choose the appropriate weighting coefficient β. The soft threshold function of Pick in the WGCNA software package was used to calculate β. Topology analysis showed that, when the threshold was β = 18, the scale-free topology fitting index (R2) was close to 0.85. This indicates that the network was close to a scale-free network (**Figure 2**). Thus, we used β = 18 as the soft threshold for constructing a co-expression network. Using the WGCNA package in R, the genes with similar expression patterns were divided into modules; 50 was set as the minimum number of genes in the module, and the threshold of cutting height was 0.25. The gene modules were identified using the dynamic cut tree method, and the modules with high similarity were combined to obtain 21 modules (**Figure 3**). Because of the large number of genes, the displayed network was difficult to identify in detail. The association diagram between modules was drawn, and the proximity of different color modules and the correlation of genes between modules were evaluated (**Figure S1**). At the same time, a visual gene network was also drawn to show the degree of association of specific genes (**Figure S2**). After generating the characteristic gene map for each module, the correlation between the characteristic genes and SCN stress was analyzed. The module most related to SCN stress was the turquoise module, with 632 DEGs, the correlation was 0.86, and the p-value was less than 0.001 (**Figure 4**).

### 3.3 Analysis of the co-expression network of the hub gene in the turquoise module

**Figure 5:**
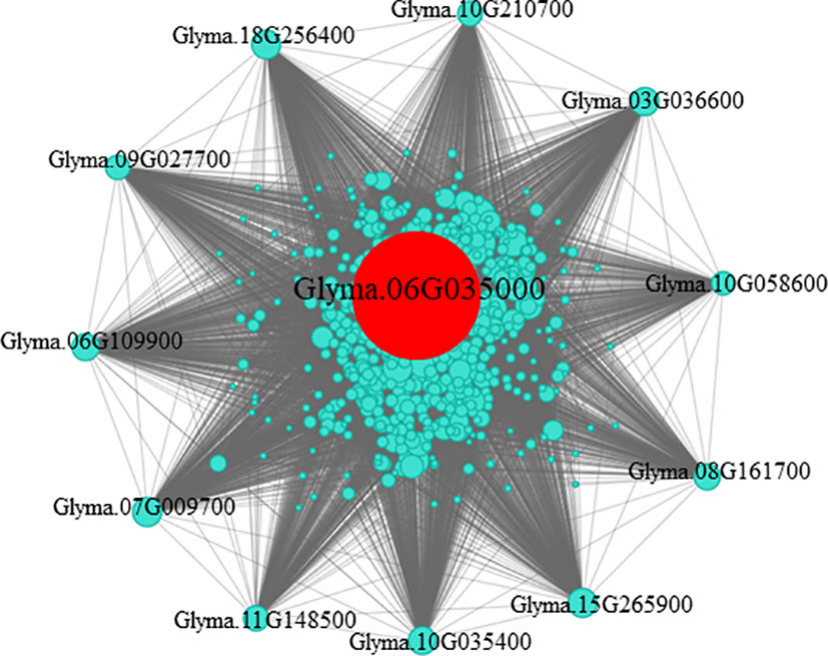
Co-expression network analysis of the hub gene in the turquoise module. The network of highly connected genes in the turquoise module. Red represents the hub gene.

In the gene co-expression network, highly connected genes are called central genes, and central genes play an important role in response to stress (Koido et al., 2018). We selected genes with an MM > 0.85 and GS > 0.6 in module turquoise (**Figure S3**), introduced them into Cytoscape, and screened them using the MMC algorithm, and selected the gene with the highest connectivity as the hub gene. The central gene *GmHg1* was found in module turquoise, and the co-expression network of the hub gene *GmHg1* was constructed (**Figure 5**). The hub gene *GmHg1* (Glyma.06G035000) encodes serine/threonine protein kinase. At the same time, 11 genes that were closest to the hub gene expression trend were found (**Table 1**). They included Glyma.09G027700 (wall-associated receptor kinase-like 1), Glyma.06G109900 (C_2_H_2_-like zinc finger protein), Glyma.07G009700 (Hydroxyproline-rich glycoprotein family), Glyma.11G148500 (Arabinogalactan-protein 11), and Glyma.10G035400 (Basic helix-loop-helix (bHLH) DNA-binding superfamily protein). The domains of these genes have been identified as being involved in plant disease resistance or abiotic stress responses (Simon et al., 2005; Lamport et al., 2006; Meier et al., 2010; Wang et al., 2019; Hao et al., 2021). Genes with the same expression trend may have the same function. It is speculated that these genes may be involved in soybean resistance to SCN.

**Table 1:** MCC algorithm result.

Gene	Score	Annotation
Glyma.06G035000	128	Protein kinase family protein
Glyma.09G027700	102	wall-associated receptor kinase-like 1
Glyma.06G109900	80	C_2_H_2_-like zinc finger protein
Glyma.07G009700	66	Hydroxyproline-rich glycoprotein family
Glyma.11G148500	64	Arabinogalactan-protein 11
Glyma.10G035400	60	Basic helix-loop-helix (bHLH) DNA-binding superfamily protein
Glyma.15G265900	59	Seed storage 2S albumin superfamily protein
Glyma.18G256400	58	Cysteamine dioxygenase/Persulfurase
Glyma.10G210700	55	Phosphoglucomutase
Glyma.08G161700	57	Seed storage 2S albumin superfamily protein
Glyma.10G058600	53	ACT-like superfamily protein
Glyma.03G036600	52	Ovate family protein 2

### 3.4 Molecular cloning and bioinformatics analysis of the *GmHg1* gene

**Figure 6:**
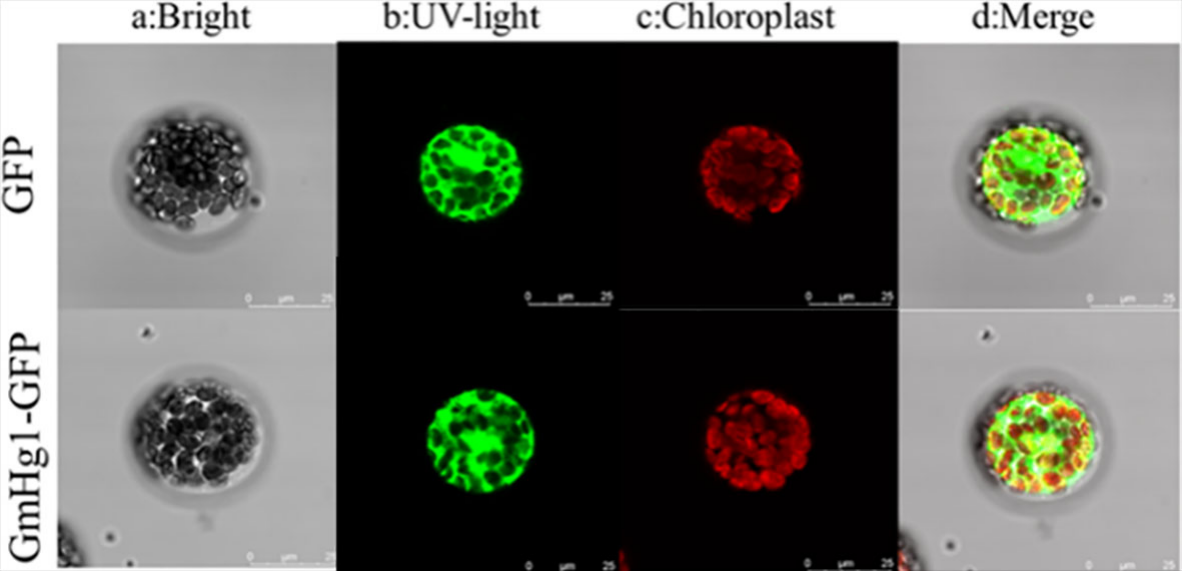
Subcellular localization of *GmHg1*.

To understand the function of *GmHg1* in soybean, we isolated the CDS fragment of *GmHg1* from ‘Dongnong L-204’. The fragments comprised 2130-bp open reading frames and were predicted to encode 710 residue polypeptides. To verify the basic characteristics of *GmHg1*, a fusion expression vector of 35S::GmHg1-GFP was constructed and transiently expressed in *Arabidopsis* protoplasts. The fusion protein was expressed in the nucleus, cell membrane, and cytoplasm of protoplasts. (**Figure 6**). At the same time, promoter element analysis of the 1000-bp upstream *GmHg1* sequence was carried out (**Table S1**). There were 16 elements in the *GmHg1* promoter sequence, among which TGACG-motif and CGTCA-motif were cis-acting elements of methyl jasmonate, which responded to abiotic stress. ABRE, ARE, and TGACG-motif elements were corresponding elements for defense and response to stress. These results suggest that *GmHg1* may be involved in resistance to stress.

### 3.5 Analysis of the expression pattern of *GmHg1*

**Figure 7:**
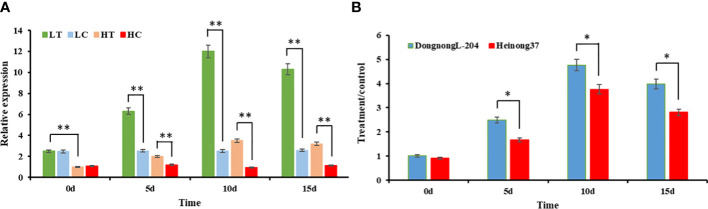
Analysis of the expression pattern of *GmHg1*. **(A)** Relative expression of *GmHg1* in resistant and susceptible varieties. LT, ‘Dongnong L-204’ treatment; LC, ‘Dongnong L-204’ control; HT, ‘Heinong 37’ treatment; HC, ‘Heinong 37’ control. **(B)** Increase in *GmHg1* expression. Asterisks indicate a significant difference compared with the corresponding control (Student’s *t*-test: **p < 0.01). Values represent the means of three biological replicates. *p < 0.05.

To clarify the expression pattern of *GmHg1* under SCN stress and verify the accuracy of transcriptome data, we treated the roots of the SCN-resistant cultivar (‘Dongnong L-204’) and the SCN-susceptible cultivar (‘Heinong 37’) with HG Type 0. The expression of *GmHg1* was determined at 0, 5, 10, and 15dpi. Before HG Type 0 induction, *GmHg1* expression in resistant varieties was higher than in susceptible varieties. After HG Type 0 induction, *GmHg1* expression in SCN-resistant/-susceptible cultivars was significantly upregulated compared with that before induction (**Figure 7**). The expression of *GmHg1* reached the highest level after 10 days of HG Type 0 stress. At the same time, the upregulation multiple in SCN-resistant cultivars was significantly higher than that in SCN-susceptible cultivars. Therefore, *GmHg1* may be involved in the resistance response of soybean to SCN, and the difference in *GmHg1* expression may lead to the difference in SCN resistance among SCN-resistant and -susceptible cultivars.

### 3.6 Identification of the disease resistance function of the *GmHg1* gene under HG Type 0 stress

**Figure 8:**
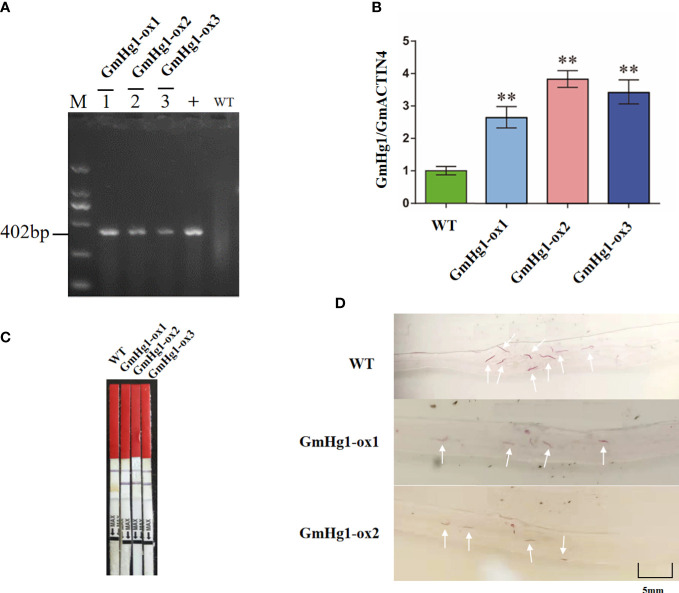
Identification of *GmHg1-OX* transgenic soybeans. **(A)** Gel image of PCR products obtained with primer sets for Bar gene regions of the vector. M: DL2000 marker. 1–3: transgenic soybeans; WT: DNA of wild-type soybean plants. +: Plasmid of the *pCAMBIA 3300*-*GmHg1* vector. **(B)** *GmHg1* gene expression level in transgenic and WT soybean by qRT-PCR. *GmACTIN4* was used as an internal reference gene. Asterisks indicate a significant difference compared with the corresponding controls (Student’s *t*-test: **p < 0.01). Values represent the means of three biological replicates. **(C)** Detection of the selectable marker gene by bar test strip. WT, wild-type soybean plants. **(D)** Hairy roots with magenta dye. The white arrow points to the soybean cyst nematode observed under 20 times magnification of the microscope.

To verify whether *GmHg1* was involved in the response to HG Type 0 stress, we used ‘Dongnong 50’ as a WT and transformed the 35S::GmHg1 recombinant vector into soybean by the semi-seed transformation method (Zhao et al., 2018). We obtained T2 generation 35S::GmHg1 overexpressing soybean and identified GmHg1-OX soybean using PCR, qRT-PCR, and bar test strips (**Figure 8**). qRT-PCR showed that the *GmHg1* expression levels were significantly increased in T2 generation plants. At the same time, we constructed a pFGC5941-GmHg1-RNAi vector, interfered with the expression of the *GmHg1* gene by RNAi, and created pure and stable soybean plants transformed with the GmHg1-RNAi vector in ‘Dongnong 50’. qRT-PCR showed that the expression levels of *GmHg1* were significantly decreased in T2-generation plants (**Figure S4**). The roots of transgenic soybean with GmHg1-OX and GmHg1-RNAi genes were further stressed by HG Type 0, and the phenotype was observed by acid fuchsin staining 10 days after inoculation (**Tables S2**, **S3**). Compared with ‘Dongnong 50’, the number of HG Type 0 in transgenic soybean plants with the GmHg1-OX gene decreased significantly from 5.34 to 2.9 per root. However, the number of cyst in transgenic soybean plants with the GmHg1-RNAi gene increased significantly from 5.34 to 7.74 per root. At the same time, cyst formation in soybean plants overexpressing the *GmHg1* gene was severely inhibited compared with that of soybean plants that interfered with *GmHg1* gene expression. The increase in *GmHg1* gene expression may improve the resistance of soybean to HG Type 0. The *GmHg1* gene plays a positive regulatory role in the process of soybean resistance to HG Type 0.

### 3.7 Expression analysis of other genes in the co-expression network in transgenic materials

**Figure 9:**
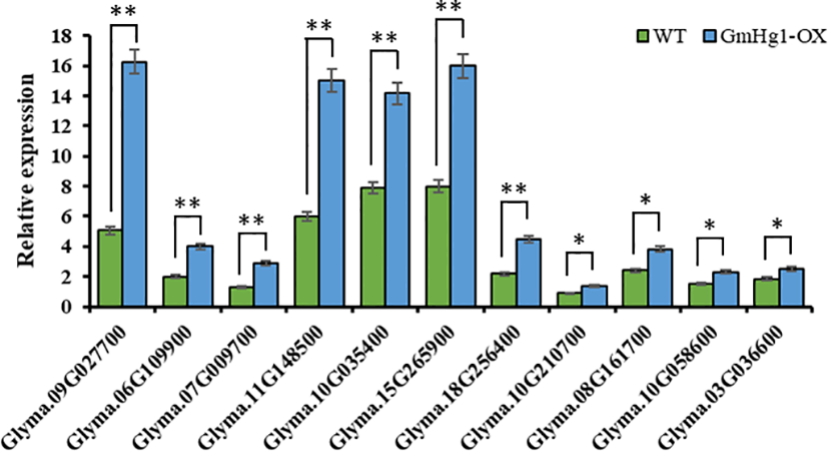
Analysis of the expression pattern of genes in the co-expression network. Asterisks indicate a significant difference compared with the corresponding controls (Student’s *t*-test: **p < 0.01). Values represent the means of three biological replicates. *p < 0.05.

To identify the expression pattern of genes similar to the hub gene expression trend in transgenic materials, we detected the expression of 11 genes in soybean seedlings transformed with GmHg1-OX and GmHg1-RNAi genes. The expression of 11 genes in GmHg1-OX transgenic soybean seedlings was significantly higher than that in ‘Dongnong 50’ (**Figure 9**). Among them, the expression of Glyma.09G027700 (wall-associated receptor kinase-like 1) increased the most, and that of Glyma.08G161700 (Seed storage 2S albumin superfamily protein) and Glyma.03G036600 (Ovate family protein 2) genes increased the least.

The expression of 11 genes in GmHg1-OX transgenic soybean seedlings was significantly lower than that in ‘Dongnong 50’ (**Figure S5**). This indicates that these 11 genes were co-expressed with *GmHg1* and may be involved in the process of soybean resistance to SCN. However, the specific resistance mechanisms of these genes and *GmHg1* need to be further studied.

## 4 Discussion

With the continuous development of second-generation sequencing technology, researchers are increasingly inclined to use high-throughput sequencing methods to study gene function. Through traditional RNA-Seq analysis, the expression changes of all genes in the sample can be obtained, but it lacks purposeful screening (Du et al., 2017). The combination of transcriptome data and the WGCNA algorithm can effectively identify the gene module of co-expression and calculate the relationship between the gene network and the phenotype concerned by the researchers (Du et al., 2017). The combination of transcriptome data and the WGCNA algorithm to mine core genes related to target traits has been widely used in plants (Luo et al., 2019). In this study, WGCNA was used to analyze the transcriptome data of soybean roots before and after HG Type 0 treatment, and a co-expression network of genes related to SCN resistance was constructed. Through systematic average linkage cluster analysis, the co-expressed genes were divided into 21 modules. The module most related to SCN stress was the turquoise module. We identified the hub gene *GmHg1* in the turquoise module and speculated that it plays an important role in soybean resistance to SCN.

Protein kinase (PK) is an enzyme that catalyzes the process of protein phosphorylation, which can catalyze the transfer of γ-phosphate groups on ATP to the amino acid residues of the substrate (Trewavas and Malho, 1997). It uses its own extracellular region to identify pathogen signals, triggers, or closes the signal transduction channels of downstream proteins through phosphorylation and dephosphorylation, regulates the function of signal transduction in plants, and participates in plant stress resistance and defense responses (Sheen, 1996). Protein kinase SOBIR1 in *Arabidopsis thaliana* plays a positive regulatory role in plant defense, indicating that PK is involved in plant stress resistance and defense responses (Gao et al., 2009). In previous studies, MAPK responded to HG Type 0 stress (Jiang et al., 2020), suggesting that protein kinase may be involved in soybean anti-SCN response, but whether it has an anti-SCN function has not been identified. In this study, transcriptome data and the WGCNA algorithm were used to mine the hub gene *GmHg1* related to SCN, which encodes a serine/threonine-specific protein kinase domain. To further verify the anti-SCN function of *GmHg1*, we obtained stable transgenic soybean seedlings overexpressing the GmHg1-OX and GmHg1-RNAi genes. As expected, compared with wild soybean ‘Dongnong 50’, the number of HG Type 0 cysts in GmHg1-OX transgenic soybean roots decreased significantly, while the number of cysts in GmHg1-RNAi transgenic soybean roots significantly increased. These results suggest that protein kinase *GmHg1* promotes soybean anti-HG Type 0 response.

The analysis of the expression pattern of *GmHg1* showed that the expression of *GmHg1* in SCN-resistant cultivars was higher than that in SCN-susceptible cultivars, and *GmHg1* expression was significantly upregulated in both resistant and susceptible varieties induced by HG Type 0, which was consistent with the expression trend of transcriptome data measured before (Jiang et al., 2020; Jiang et al., 2021). It also showed that *GmHg1* positively regulated the resistance of soybean to HG Type 0. *GmHg1* expression reached the highest level on the 10th day after HG Type 0 stress, which may be due to the fact that this stage is the key period for SCN to form syncytids in soybean (Klink et al., 2007). In the turquoise module, which was most related to SCN stress, we also found 11 genes with the same expression trend as the hub gene. Among these genes, Glyma.09G027700 (wall-associated receptor kinase-like 1) has been demonstrated to activate the expression of defense genes, such as course-related proteins, and improve plant disease resistance (Meier et al., 2010). Glyma.06G109900 encodes a C_2_H_2_-like zinc finger protein, which regulates leaf senescence and drought stress (Wang et al., 2019). Glyma.07G009700 belongs to the Hydroxyproline-rich glycoprotein family, which acts as lectins, lignin deposition sites, and structural barriers in the process of plant–pathogen interactions (Simon et al., 2005). Glyma.11G148500 encodes Arabinogalactan-protein 11, which promotes root and fiber elongation and improves plant defense ability (Lamport et al., 2006). Glyma.10G035400 encoded the bHLH DNA-binding superfamily protein; bHLH transcription factors play an important regulatory role in plant growth and development and resistance to abiotic stress, such as drought, high salinity, and low temperature (Hao et al., 2021). However, other genes have not yet been reported to be related to disease resistance, and whether these genes are related to SCN resistance needs to be further verified. In this study, we measured the expression of 11 genes in soybean seedlings transformed with GmHg1-OX and GmHg1-RNAi genes. The results showed that 11 genes were co-expressed with *GmHg1*, which further verified the accuracy of WGCNA and suggested that these genes may be involved in the process of resistance to SCN with *GmHg1*.

In summary, we mined a central gene, *GmHg1*, and verified its function for HG Type 0 resistance using transcriptome and WGCNA. Meanwhile, we can speculate that genes in the turquoise module may also be involved in the resistance response of soybean to SCN.

## 5 Conclusion

A gene encoding serine/threonine protein kinase named *GmHg1* has been identified to be associated with SCN resistance. Soybean plants transformed with GmHg1-OX had significantly increased SCN resistance. In contrast, the GmHg1-RNAi transgenic soybean plants significantly reduced SCN resistance. We found that 11 genes with the same expression trend as GmHg1 gene may be involved in the process of soybean resistance to SCN. Our study provides a new direction for clarifying the molecular mechanism of soybean resistance to SCN.

## Data availability statement

The original contributions presented in the study are included in the article/**Supplementary Material**. Further inquiries can be directed to the corresponding authors.

## Author contributions

HJ: methodology, writing-original draft, writing-review and editing. CZ: data curation. JM: data curation. SQ, FL and HS: data curation. XZ: supervision. YH: project administration, supervision, writing-review and editing. All authors contributed to the article and approved the submitted version.

## Supplementary material

Supplementary Table 1 Analysis of promoter elements of *GmHg1.*

Supplementary Table 2 Average number of female plants(cm^-1^). P is for plant.

Supplementary Table 3 Paired samples t-test results.

Supplementary Table 4 Primer list.
