# Supplementary material for: Weighted gene co-expression network analysis identifies genes related to HG Type 0 resistance and verification of hub gene GmHg1
Source: Front Plant Sci. 2023 Jan 27;13:1118503. doi: 10.3389/fpls.2022.1118503 (PMC9911859; doi:10.3389/fpls.2022.1118503)
Supplement: Supplementary file 2 [file DataSheet_1.docx]

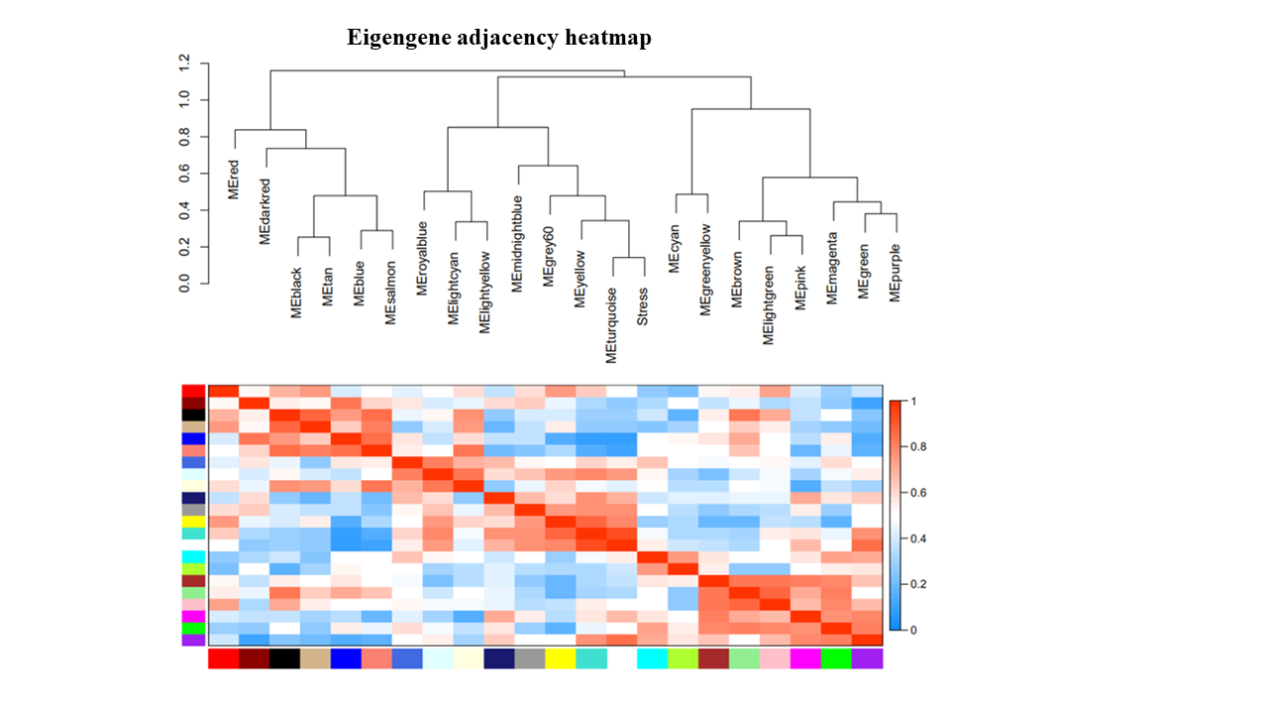


Figure S1. Hierarchical clustering dendrogram of module eigengenes and heatmap plot of the adjacencies in the eigengene network (labeled by their colors). In the heatmap, green represents low adjacency (negative correlation), while red represents high adjacency (positive correlation).


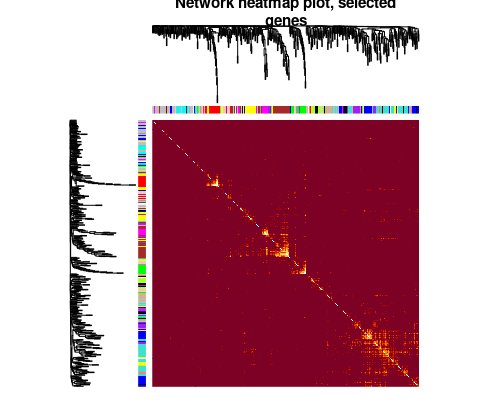


Figure S2. Weighted correlation network analysis (WGCNA) of the expression profiles of all genes. Genes were clustered by expression patterns, as represented by the dendrogram and correlation heat map. Clusters of genes are referred to as modules by color. In the heat map, the intensity of red coloring indicates the strength of correlation between pairs of genes on a linear scale. Genes that could not be assigned to a module are gray.


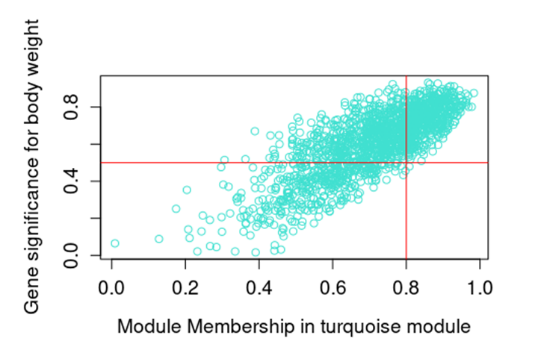


Figure S3 Significance of genes in modules


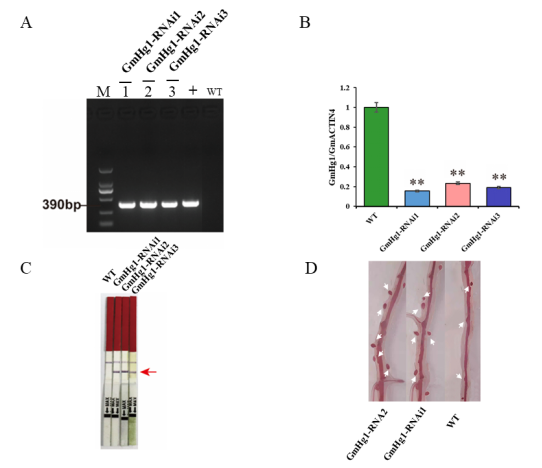


Figure S4 Identification of GmHg1-RNAi transgenic soybeans. (A) Gel image of PCR products obtained with primer sets for T-DNA regions of vector. M：DL2000 marker. 1-3: transgenic soybeans; WT: DNA of wild-type soybean plant. +: Plasmid of the pFGC5941-GmHg1-RNAi vector. (B) *GmHg1* gene expression level in transgenic soybean and WT soybean by qRT-PCR. *GmACTIN4* was used as internal reference gene. Asterisks indicate a significant difference compared with the corresponding controls (Student’s *t*-test: ***p* < 0.01). Values represent the means of three biological replicates. (C) Detection of the selectable marker gene by bar test strip. WT: wild-type soybean plants. (D) Hairy roots with magenta dye. The white arrow points to the soybean cyst nematode observed under 20 times the microscope.


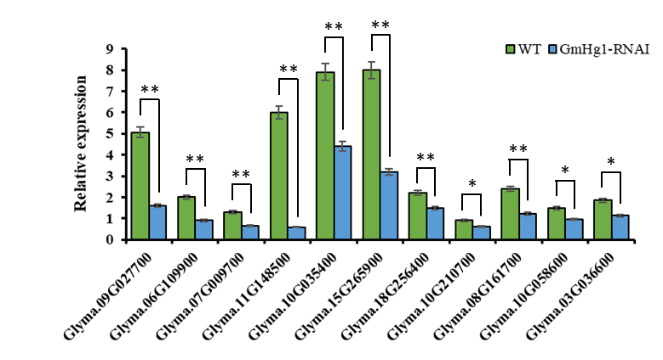


Figure S5 Analysis of expression pattern of genes in co-expression network
